# Clinical and Surgical Outcome After Oncovascular Surgery of Soft Tissue and Osteogenic Sarcomas of the Limbs

**DOI:** 10.1002/cnr2.70353

**Published:** 2025-10-08

**Authors:** Sebastian Kapahnke, Matthias Bürger, Melanie Rusch, Grischa Hoffmann, Philipp Johannes Pauli, Lars Hummitzsch, Martin Albrecht, Roland Bertolini, Julia Bertolini, Rene Rusch, Rouven Berndt, Christoph Röcken, Daniel Drücke, Katharina Hess

**Affiliations:** ^1^ Clinic of Vascular and Endovascular Surgery, University Hospital of Schleswig‐Holstein Kiel Germany; ^2^ Department of Anesthesiology and Intensive Care Medicine University Hospital of Schleswig‐Holstein Kiel Germany; ^3^ Clinic for Orthopedics and Trauma Surgery, Department for Hand‐, Plastic‐ and Microsurgery, Sarcoma‐Center University Hospital of Schleswig‐Holstein Kiel Germany; ^4^ Clinic of Vascular Medicine, University Heart and Vascular Center Hamburg, University Medical Center Hamburg‐Eppendorf Hamburg Germany; ^5^ Department of Pathology University Hospital Schleswig‐Holstein, Campus Kiel Kiel Germany; ^6^ Institute of Clinical Research and Systems Medicine, HMU—Health and Medical University Potsdam Germany

**Keywords:** oncovascular surgery, sarcoma, tumor, vascular graft, vascular reconstruction, vascular surgery

## Abstract

**Background:**

Soft tissue sarcomas (STS) and osteogenic sarcomas (OGS) of the limbs are rare diseases. Nowadays, most patients with STS or OGS undergo tumor resection and subsequent vascular reconstruction for potential limb preservation.

**Aims:**

Due to very limited data on these complex surgical procedures, the aim of this single‐center, retrospective study was to evaluate the surgical and oncological outcomes of these patients.

**Methods:**

From 2013 to 2023, demographic, clinical, surgical, and pathological data regarding tumor disease, surgical treatment, and postoperative care of a total of 10 patients with STS and OGS were identified and analyzed. Furthermore, overall survival (OS) and freedom from tumor recurrence (FFT) were retrospectively investigated among all patients.

**Results:**

The mean age of the patients was 64.4 ± 22.24 years, and six women (60%) and four men (40%) were treated. Overall, 16 major arterial and venous vessels were resected and reconstructed: the lower extremity was affected in nine patients (90%). Autologous veins (*n* = 12, 75%), polytetrafluoroethylene (PTFE; *n* = 2, 12.5%), or cryopreserved allografts (*n* = 2, 12.5%) were mainly used for vascular reconstruction. The follow‐up ranged from 7 to 60 months, with a median OS of 48 months and a median FFT of 54 months. Overall, four patients (40%) developed local tumor recurrence at the primary surgical resection site or metastasis. The primary graft patency for all vascular reconstructions was 90% at the median follow‐up of 24 months. All revascularized limbs among these patients could be salvaged during the follow‐up period.

**Conclusion:**

Treatment of patients with STS or OGS of the limbs and subsequent vascular reconstruction can be performed safely and effectively. The outcomes described in this cohort suggest that an interdisciplinary team, including vascular surgeons and a carefully planned and rigorous clinical approach, might positively influence the postoperative and oncological outcome and limb salvage.

**Abbreviations**
ACT: activated clotting time
ASA: American Society of Anesthesiologists
BMI: body mass index
DOAC: direct new oral anticoagulants
FFT: freedom from tumor recurrence
G: grading
ICU: intensive care unit
IR: ionizing radiation
IRFS: intraoperative rapid frozen sections
LS: limb salvage
OGS: osteogenic sarcoma
OS: overall survival
PTFE: polytetrafluorethylene
QoL: quality of life
RCX: radiochemotherapy
STS: soft tissue sarcomas
UCCSH: University Cancer Center Schleswig‐Holstein

## 1 Introduction

Primary malignant tumors of the musculoskeletal system are a rare solid neoplastic entity with an incidence of less than 1% in adults [1, 2]. Essentially, soft tissue sarcomas (STS) and osteogenic sarcomas (OGS) can occur in any region of the body. However, the most frequent localization is the lower extremities, with up to 50% of all cases [3, 4]. Currently, curative therapy includes surgical en bloc tumor resection combined with an individualized decision for neoadjuvant or adjuvant radiochemotherapy [5]. Historically, vascular infiltration was usually considered to be a contraindication for curative resection, resulting in major limb amputation as the only remaining therapeutic option [6]. Nowadays, the treatment of these malignancies requires a multidisciplinary therapeutic approach to aspire to a limb‐sparing surgical and oncological strategy and subsequent good quality of life (QoL). Accordingly, the involvement of vascular surgeons in the multidisciplinary team is often required for the reconstruction of venous and arterial vessels. Data in the field of oncovascular surgery are extremely limited, and clear standards for reconstruction techniques and potential graft materials have not been defined in terms of guidelines so far [7]. Moreover, mid‐term and long‐term results of surgical and oncological outcomes in patients with STS and OGS of the extremities have barely been reported [6, 7]. The majority of studies in the field of oncovascular surgery of the extremities are limited by small sample sizes and exhibit substantial heterogeneity with respect to tumor histology, types of vascular grafts utilized, and reported occlusion rates [6, 7, 8, 9, 10, 11, 12, 13, 14, 15]. Therefore, the aim of this retrospective, single‐center study was to evaluate the clinical and surgical outcomes of patients who underwent vascular reconstruction after en bloc resection of STS and OGS, with a particular focus on graft material and perioperative complications due to vascular surgery.

## 2 Materials and Methods

### 2.1 Ethical Approval

The study was approved by the local ethics committee of the University Medical Center Schleswig‐Holstein, Kiel, Germany (protocol identification number: D463/24), and all procedures, analyses, and data proceedings were performed in accordance with the Helsinki Declaration of 2013.

### 2.2 Oncological and Surgical Management

**FIGURE 1:**
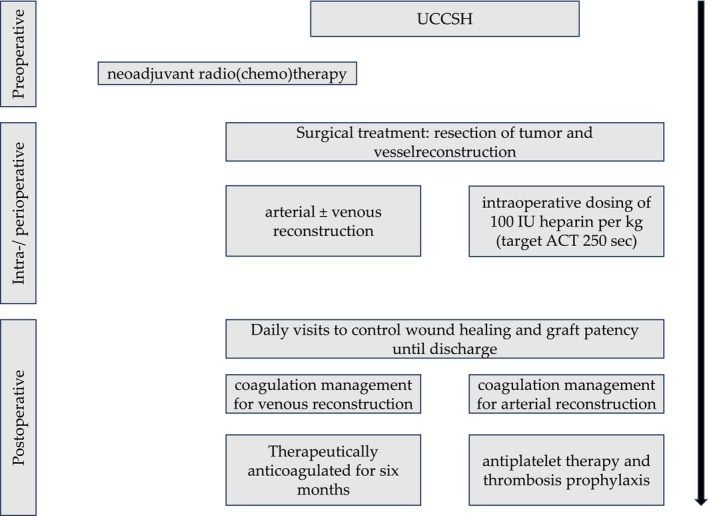
Depiction of patients' treatment timeline divided into preoperative, intraoperative, and postoperative sections. In all cases, the University Cancer Center Schleswig‐Holstein (UCCSH) determines if neoadjuvant radio(chemo)therapy treatment will be performed before scheduled surgical resection during the preoperative period. Intraoperatively, it was indicated whether vessels had to be resected and reconstructed. During reconstruction, a temporary ACT (activated clotting time) of 250 s was achieved by weight‐adapted heparin administration. Close clinical checks were carried out on wound conditions and bypass patency in the postoperative phase. In addition, anticoagulation was adjusted depending on the vascular reconstruction.

All patients have been referred to the University Cancer Center Schleswig‐Holstein (UCCSH) and subsequently presented and discussed at the local sarcoma board, including oncologists, radiation therapists, pathologists, and general, plastic, and vascular surgeons. After the initial determination of treatment (operable vs. non‐operable and adjuvant vs. neoadjuvant therapy), patients underwent biopsy, neoadjuvant radio(chemo)therapy, or initial surgical treatment. After the initial determination of the diagnosis by histopathology and molecular pathology, further treatment options, including surgery and/or adjuvant radio(chemo)therapy, were defined by the sarcoma board. A consensus decision of the sarcoma board was necessary for further proceeding (Figure 1). Neoadjuvant radiochemotherapy was applied on a case‐by‐case basis for the reduction of tumor volume before surgical resection.

Oncovascular surgery was performed by a multidisciplinary team, and complete vascular reconstruction of all affected vessels was aspired. Autologous veins, or alternatively cryopreserved allografts, were generally the preferred graft materials. Intraoperative rapid frozen sections (IRFS) analysis was performed to achieve pR0 resection. Adjuvant radiotherapy or radiochemotherapy was applied to all patients of the current cohort. The first control visit after discharge was scheduled after 2 weeks and included the control of graft patency, wound healing, and the beginning of rehabilitation. The patency of the vascular reconstructions was verified using duplex sonography or contrast‐enhanced computed tomography. This was performed immediately after surgery, after 2 weeks, and then at intervals of 4–12 weeks on a case‐by‐case basis. The functionality of the treated limb was also examined at each follow‐up appointment.

### 2.3 Data Collection and Study Design

**FIGURE 2:**
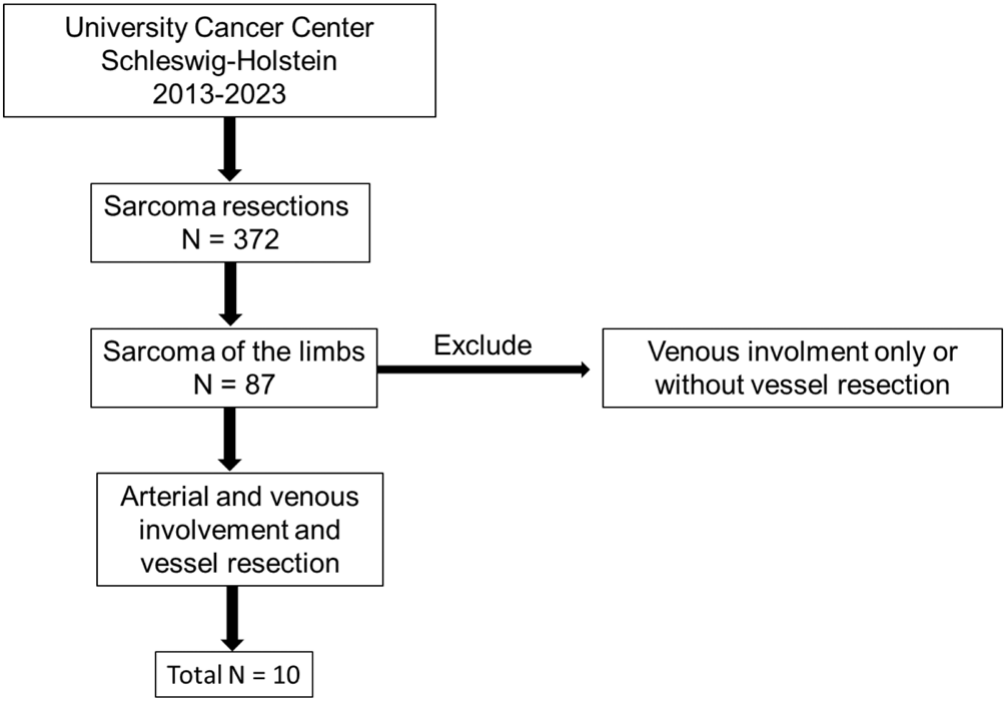
Flow chart of the selection of the study cohort.

The derivation of the study cohort is depicted in Figure 2. All data were obtained from the UCCSH to identify patients who underwent tumor excision due to STS or OGS of the limbs with en bloc arterial and venous resection and at least arterial reconstruction between 2013 and 2023. Patients with isolated venous involvement were excluded due to the not routinely performed reconstruction of veins in the setting of major tumor resections. Patient data were retrieved from the electronic medical records of the UCCSH. Overall, 10 patients were identified and included in the retrospective study presented here.

Diagnosis was confirmed in all cases by preoperative biopsies and/or from the surgically resected specimens. The extent of tumor involvement of the neurovascular bundle was defined by using standard imaging techniques (magnetic resonance imaging, computed tomography, and sonography). Data on patient demographics, relevant comorbidities, and oncological details (e.g., tumor localization, tumor classification, concomitant radiotherapy and/or chemotherapy) were recorded and analyzed.

Collected perioperative data included time of surgery, blood loss during surgery, mean cross‐clamping time, and pathological assessment of the resection margin (R0—no cancer cells seen microscopically, R1—cancer cells seen microscopically, R2—macroscopic residual tumor seen). Data on vascular reconstruction included the affected vessel(s) and the graft material used for reconstruction (autologous, allogenic, alloplastic). The evaluated outcome data included all surgery‐related complications, such as postoperative bleeding, wound infection, graft thrombosis, and clinical signs of persistent or temporary malperfusion. Furthermore, overall survival (OS), freedom from tumor recurrence (local or metastases) (FTT), and limb salvage (LS) were observed among all patients.

### 2.4 Statistical Analysis

Clinical endpoints were defined as OS, FFT, and LS. Descriptive statistics were employed for data analysis. Categorical variables were reported as absolute counts (*n*) and percentages (%), and continuous variables as central tendency and variation (mean ± standard deviation, median with range). OS and FFT follow‐up were analyzed using the Kaplan–Meier method. Statistical analysis was performed with the Graph Pad Prism version 9.2.0 (Graph Pad Software; San Diego, USA).

## 3 Results

### 3.1 Patient Demographics and Tumor Entities

A total of 10 patients were treated for STS or OGS of the upper or lower extremities and concomitant vascular reconstruction in the UCCSH between 2013 and 2023. The mean age of patients was 64.4 ± 22.24 years, with a slightly higher number of females (*n* = 6; 60%) compared to males (*n* = 4; 40%). Diagnosed tumor entities comprised of osteosarcoma (*n* = 3; 30%), synovial sarcoma (*n* = 3; 30%), and undifferentiated pleomorphic sarcoma (*n* = 4; 40%) (Table 1). Pathological R0 tumor resection was achieved in all patients of the cohort without residual tumor cells in the histological analysis of the resected tissue margin. In addition, histological analysis of all patients confirmed a pV0 status (no tumor cells within resected vessels), as presented in Table 1.

**TABLE 1:** Localization and histological characteristics of the resected tumors [*n*, (%)] and grading (G) according to the classification of the FNCLCC defined by a combination of tumor differentiation, mitotic count, and necrosis. Vascular infiltration (V) according to the TNM classification [16].

Entity	Number (%)
Osteosarcoma	3 (30%)
Upper arm	1
G2, V0	1
Upper leg	2
G1, V0	1
G3, V0	1
Synovial sarcoma	3 (30%)
Upper leg	3
G2, V0	2
G3, V0	1
Undifferentiated pleomorphic sarcoma	4 (40%)
Upper leg	2
G3, V0	2
Groin	2
G3, V0	2

### 3.2 Surgical Data

Oncovascular surgery was performed by an interdisciplinary team including oncological, plastic, and vascular surgeons. As prior approved by the interdisciplinary sarcoma board, the main goal was to perform en bloc resection of the tumor with subsequent limb‐preserving reconstruction of the major arterial and venous vessels. Tumor localization and major vessel infiltration included vessels of the groin/upper leg in nine (90%) and upper extremity in one (10%) patient, respectively.

**FIGURE 3:**
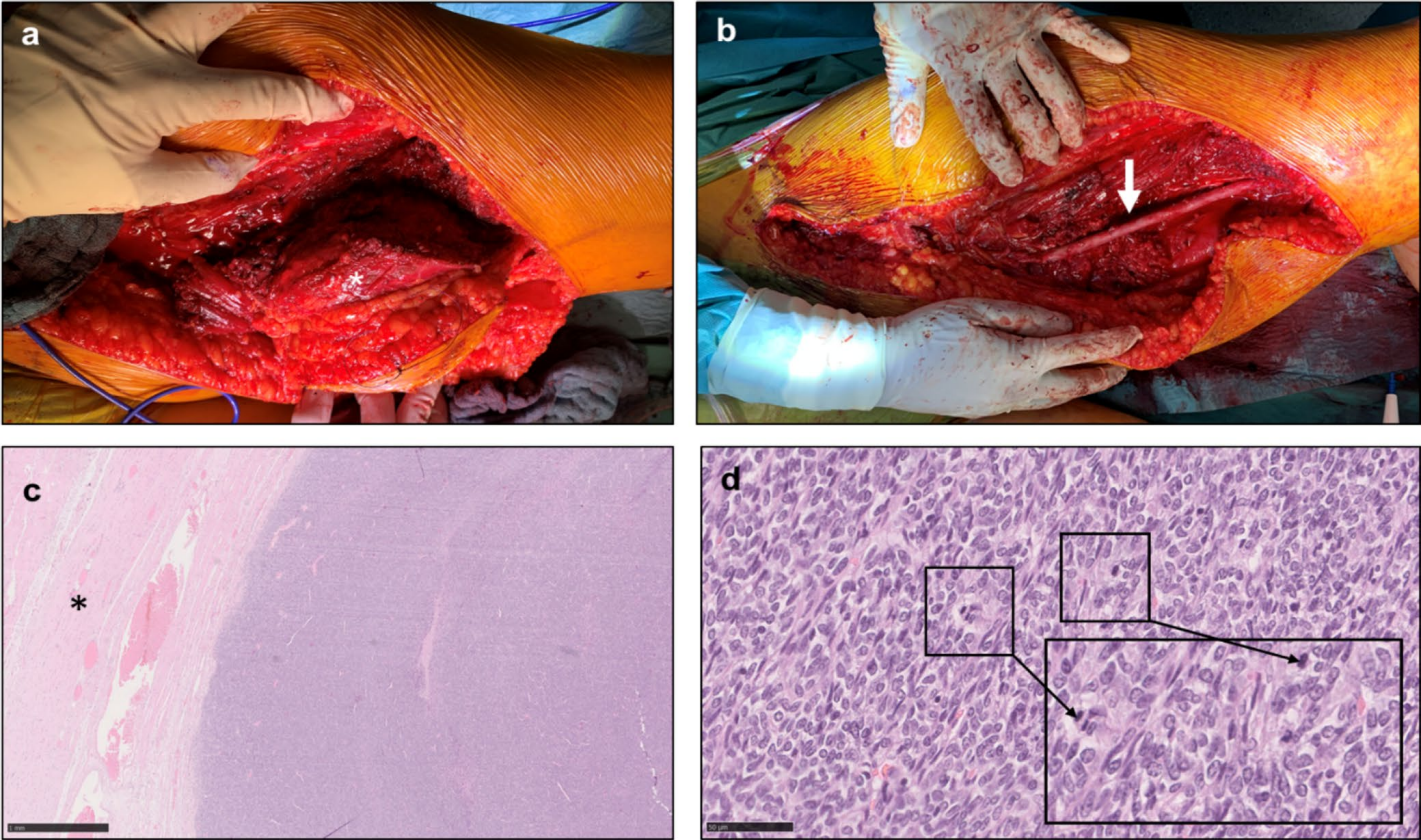
Depiction of a synovial sarcoma (white asterisk) of the upper leg (a) and reconstruction of the superficial femoral artery via a cryopreserved donor graft (white arrow) (b). Histomorphology of the synovial sarcoma: large, solid tumor (c) was removed, relatively sharply demarcated from the surrounding muscle tissue (black asterisk; magnification 25×). The tumor consists of spindle‐shaped, polymorphic cells with size‐variant nuclei and numerous mitoses (black arrows, inlet as 400× magnification) (d).

In the current cohort, a total of 10 major arteries and 10 veins were resected. Arterial resections involved the brachial, superficial femoral, or iliac arteries, while venous resections included either the femoral or brachial veins (Table 2). In general, the use of biological materials for vascular reconstruction was both preferred and aspired to. If arterial and venous reconstruction was necessary and suitable autologous material was available, both vessels were reconstructed using autologous veins. If no suitable autologous material was available, a suitable allograft was considered and implanted. If no autologous material or allograft was available, arterial and venous reconstruction was performed with PTFE (Table 3). Artificial material did not limit the extent of vessel reconstruction in general. However, reconstruction of resected femoral veins was limited due to technical reasons, the degree of tissue damage, and general considerations, for example, intraoperative critical conditions of the patient. All arteries were successfully reconstructed and replaced, whereas only 6 of the 10 veins (60%) underwent replacement using various graft materials. Most commonly, the resected and infiltrated vessels were reconstructed using autologous veins (arterial replacement: *n* = 7; venous replacement: *n* = 5) or cryopreserved donor grafts (arterial replacement: *n* = 1; venous replacement: *n* = 1), as illustrated in Figure 3. In selected cases, PTFE prostheses were implanted for vessel replacement (*n* = 2) due to the unavailability of suitable allogenic or autologous grafts. Table 3 provides a detailed overview of the materials used for reconstruction.

**TABLE 2:** Overview of resected vessels.

Resected vessels	Number (%)
Arterial system	10 (100%)
Brachial artery	1 (10%)
Superficial femoral artery	8 (80%)
Iliac artery	1 (10%)
Venous system	10 (100%)
Femoral vein	9 (90%)
Brachial vein	1 (10%)

**TABLE 3:** Overview of materials used for reconstruction.

Material used for reconstruction	Number (%)
Arterial system	10 (100%)
Autologous vein	7 (70%)
Cryopreserved donor grafts	1 (10%)
PTFE	2 (20%)
Not reconstructed	0 (0%)
Venous system	10 (100%)
Autologous vein	5 (50%)
Cryopreserved donor grafts	1 (10%)
Not reconstructed	4 (40%)

In vascular reconstruction, plastic surgical defect coverage with complex reconstructive procedures such as free flap surgery with microvascular anastomosis is often not necessary based on the experience at UCCSH. This is primarily due to the pre‐stretched tissue caused by the tumor and the subfascial location, which allows parts of the skin and subcutaneous fat tissue to be spared and utilized as local flaps. Furthermore, the tumor resection itself creates an area that facilitates easier closure.

Preservation of the surrounding tissue in combination with the subfascial location of the tumor allows for primary closure of the surgical cavity.

### 3.3 Perioperative and Postoperative Management

All patients were initially introduced to the UCCSH and the institutional sarcoma board, including oncologists, radiation therapists, pathologists, and general, plastic, and vascular surgeons. After initial determination of treatment (operable vs. non‐operable and adjuvant vs. neoadjuvant therapy), patients underwent biopsy, neoadjuvant radio(chemo)therapy, or initial surgical treatment. After initial determination of the diagnosis by histopathology and molecular pathology, further treatment options, including surgery and/or adjuvant radio(chemo)therapy, were defined by the sarcoma board (Figure 1). Depending on tumor size and location, lipocutaneous or fasciocutaneous skin flap surgery was avoided. The first control visit after discharge was scheduled after 2 weeks and included the control of graft patency, wound healing, and the beginning of rehabilitation.

After surgery, the patients stayed at the hospital for 9.5 ± 3.01 days before being discharged home or to a rehabilitation facility. Perioperative characteristics are summarized in Table 4.

**TABLE 4:** Perioperative data of treated patients.

Perioperative characteristics	
ASA score	1.9 ± 0.49
BMI (kg/m^2^)	23.79 ± 15.44
Operation time (min)	175.9 ± 78.98
Blood loss during surgery (mL)	640 ± 373.36
Blood transfusion (units of red blood)	0.7 ± 0.61
Cross‐clamping time (min)	49 ± 10.81
ICU stay (days)	1.3 ± 1.21
Hospital stay (days)	9.5 ± 3.01

Abbreviations: ASA, American Society of Anesthesiologists; BMI, body mass index; ICU, intensive care unit.

Furthermore, four patients received adjuvant radiotherapy, six patients underwent neoadjuvant radiotherapy, and three patients received additional neoadjuvant radiochemotherapy, as decided by the interdisciplinary sarcoma board.

One patient demonstrated a sensory deficit in the upper leg subsequent to the reconstruction of the femoral artery and vein. Another patient experienced a sensory deficit of the hand after reconstruction of the brachial artery of the corresponding arm. Both symptoms diminished after 4 and 6 months, respectively. In addition, one wound and graft infection (cryopreserved donor grafts) after 8 weeks and one thrombotic occlusion (after 7 months, involving reconstruction of the femoral artery and vein with an autologous vein) were observed as major complications. Postoperative complication management included successful surgical embolectomy due to limb ischemia and thrombotic occlusion in one patient after 7 months, and, in addition, one patient received open wound treatment and consecutive vacuum therapy due to wound infection after 6 months (Table 5). LS was achieved in all patients. Assessing and documenting the functionality of the treated limb was performed during each follow‐up visit. Apart from the two temporary sensory deficits described above, functionality of the limbs was preserved in all treated patients.

**TABLE 5:** Overview of postoperative vascular graft‐related complications and outcome. Primary patency was defined as patency during the interval between primary vascular surgery and repeated intervention.

Patients	Vascular reconstruction	Major postoperative complications	Primary patency
1	PTFE	—	24 months
2	Vein	Thrombotic occlusion and successful embolectomy (7 months)	7 months
3	Vein	—	60 months
4	Allograft	—	24 months
5	Vein	—	19 months
6	Vein	—	48 months
7	Vein	Wound and graft infection (6 months) and successful Open wound treatment with vacuum therapy	8 months
8	Vein	—	18 months
9	PTFE	—	24 months
10	Vein	—	7 months

Abbreviation: PTFE, polytetrafluoroethylene.

### 3.4 Regime of Anticoagulation

Oncovascular surgery was performed in an average time of 175.9 ± 78.98 min. The average cross‐clamping time for vascular reconstruction was 49 ± 10.81 min. Each patient lost an approximate mean of 640 ± 373.36 mL of blood during surgery. For this purpose, all patients received transfusions of an average of 0.7 ± 0.61 red blood cell concentrates. The average intensive care unit (ICU) stay was 1.3 ± 1.21 days. Patients undergoing reconstruction of resected venous segments were therapeutically anticoagulated with fractionated and unfractionated heparin postoperatively and later switched to phenprocoumon or direct oral anticoagulants (DOAC) for at least 6 months. In contrast, patients who underwent arterial reconstruction received only antiplatelet therapy and thrombosis prophylaxis with unfractionated heparin. However, any decision regarding coagulation management was made based on interdisciplinary consensus and individual, patient‐specific risk management (Figure 1).

### 3.5 Follow‐Up and Patency of Reconstructed Vessels

**FIGURE 4:**
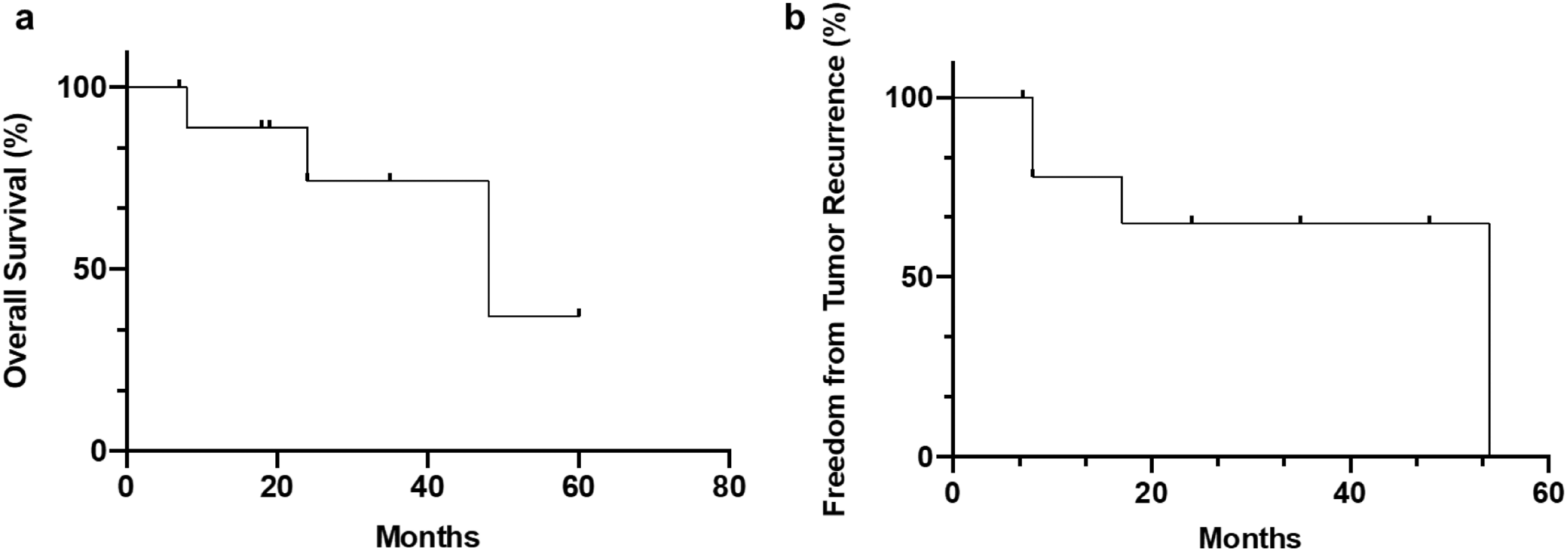
Cumulative Kaplan–Meier curve of overall survival (OS) (a) and freedom from tumor recurrence (FFT) (b).

The median follow‐up duration was 24 months, ranging from 7 to 60 months, with a median OS of 48 months (refer to Figure 4a). The first tumor recurrence was observed in two patients after 8 months; overall, three patients (30%) developed local tumor recurrence at the primary surgical resection site (radiologically proven tumor‐suspicious neoplasia during follow‐up). One patient (10%), diagnosed with a synovial sarcoma, experienced lung metastasis 17 months post‐follow‐up. Accordingly, the median FFT within this cohort was 54 months (see Figure 4b).

No patient underwent a second operation due to tumor recurrence. Three patients (30%) died during follow‐up due to pneumonia, sepsis of unknown cause, or myocardial infarction.

Regarding vascular reconstructions, the primary graft patency rate was found to be 90% at the median follow‐up of 24 months. One patient required a second embolectomy for the arterial and venous reconstruction 7 months postoperation, after which no further graft occlusions were noted. Throughout the follow‐up period, LS was successfully achieved for all revascularized limbs in these patients.

## 4 Discussion

Soft tissue and bone sarcomas represent a relatively rare tumor entity in adults affecting the extremities, yet they pose a significant challenge to various medical specialties—particularly to oncovascular surgery. To the best of our knowledge, the available studies are based on small, heterogeneous patient cohorts that include varying tumor locations and diverse surgical approaches [7, 9, 14, 15]. The lack of comprehensive data on extremity sarcomas and oncovascular surgery may be explained by the relatively low prevalence of STS and OGS, as well as the current trend toward multidisciplinary reconstructive therapies that emphasize limb preservation [9, 10, 13]. Our interdisciplinary approach highlights the positive results of reconstructive surgery in tumor resections [17, 18, 19]. The demographic data of our study participants were diverse, with no apparent correlations between patient outcomes and age or gender. The age of the patients differed widely and ranged from 22 to 88 years, with a tendency toward older patients. This aligns with findings from Cruz et al., Kekeç et al., Spark et al., Obi et al., and Nishinari et al., and underlines the need for a highly individualized and multidisciplinary therapeutic approach [10, 13, 20, 21, 22].

In accordance with previous literature [11, 13, 23, 24], most of the cases of STS and OGS presented here involved the lower extremities, particularly the thighs. Our surgical approach focused on en bloc and pR0 resections, followed by vascular reconstruction of infiltrated vessels, in line with current recommendations [7, 10, 25, 26]. Achieving pR0 resection in all patients was possibly aided by the consistent and consequent use of IRFS during surgery.

The sarcoma patients were treated according to the ESMO–EURACAN–GENTURIS (European Society for Medical Oncology; European Reference Network for Rare Adult Solid Cancers; European Reference Network for Genetic Tumour Risk Syndromes) guidelines for the treatment of soft tissue and bone sarcomas [27, 28]. As recommended, all patients were presented to an interdisciplinary tumor board that included at least one surgical specialty, oncology, pathology, radiology, and radiotherapy. Localization‐specific expertise was consulted on a case‐by‐case basis. Follow‐up examinations are performed with MRI or CT within 3–6 months in the first and second years, every 6 months in Years 3–5, and then annually after 6 years. Complete resection is recommended as the primary treatment goal for patients; tumor‐free margins with a wide safety margin should be achieved [27, 28].

Nevertheless, there is an ongoing debate on the impact of pR0 resection and adequate resection margin on prognosis and tumor‐free survival in patients with STS and OGS [29, 30]. Former studies had usually focused on radical surgical approaches and the definition of the optimal surgical resection margin to improve OS [29, 30]. In contrast, recent studies and meta‐analyses have demonstrated that only R0 resection, but more importantly, the quality of surgical margins and therefore tumor biology, might have a relevant impact on the patient's outcome. Hence, Harati and Lehnhardt stated that a radical surgical approach with the goal of wide negative margins could not be justified by the available study data [30]. Our study's observation of local recurrence or metastasis in four cases, despite predominantly R0 resections, supports this argumentation and suggests that complete macroscopic and microscopic removal does not entirely eliminate recurrence risks in sarcomas [31]. IRFS analysis might potentially improve resection quality; its role in sarcoma surgery requires further clarification. Surgical margin classification, as described by Gundle et al., might aid in assessing recurrence risks and guiding multimodal therapy [32].

A frequently discussed aspect is the use of various graft materials for vascular reconstruction. There is a lack of clinical trials systematically comparing the advantages and disadvantages of autologous, allogeneic, xenogeneic, or artificial grafts and associated coagulation protocols in oncovascular patients [23, 24, 33, 34]. The impact of adjuvant therapies, like radiochemotherapy, on vascular graft reconstruction is not well understood.

However, there is a general consensus on the harmful effects of ionizing radiation (IR). There are two mechanisms: a direct mechanism (damage to cellular deoxyribonucleic acid) and an indirect mechanism (formation of free radicals) [35, 36]. IR can lead to endothelial dysfunction, increased inflammatory mediators, vascular hyperpermeability, and fibrosis of the affected tissue [37, 38]. Virmani et al. and Stewart et al. were able to demonstrate an enlarged media–intima complex inside the vessel after radiation exposure, similar to atherosclerotic disease [39, 40]. Previous studies have shown that patients treated with radiotherapy for a nasopharyngeal tumor have a higher incidence of stenosis of the carotid artery than persons not exposed to radiation [41, 42, 43]. In the present study, no significant tissue alterations, according to the surgical protocols, were identified that might have influenced the surgical outcome. It should be noted, however, that the size of the patient cohort is too limited to allow for statistically meaningful conclusions. Further research involving larger, prospective patient cohorts is warranted to adequately investigate these potential effects. There are currently no prospective clinical studies on vascular graft complications under adjuvant radiotherapy, and therefore, the ideal graft type has not been identified so far in this distinct cohort.

In the current study, the authors preferred the autologous great saphenous or femoral vein as the primary graft due to previously described good long‐term patency rates and the prevention of bacterial infection [17, 44, 45, 46, 47]. In cases of unavailability of sufficient autologous veins, the authors opted for reconstruction using cryopreserved human vessels. Iliac arteries were routinely replaced with polyester grafts to prevent anastomosis mismatches based on well‐known favorable patency rates of large‐diameter alloplastic grafts. Thus, an excellent graft patency of 90% at the median observation period of 24 months was assessed in the study cohort presented here. However, the authors concluded that the thrombotic occlusion of the femoral reconstruction great saphenous vein after 7 months can be explained by the fact that therapeutic anticoagulation (in this case, phenprocoumon) was switched to single antiplatelet therapy (acetylsalicylic acid) after 6 months in the case of spontaneous intestinal bleeding.

Schwarzbach et al. showed a limb preservation of 94.1% in their cohort of patients observed with a median follow‐up of 30.4 months [48]. In contrast, Teixeira et al. reported an amputation rate of 38.46% after previous vascular reconstruction in a cohort of 13 patients and highlighted the presence of bone sarcoma or surgical site infection as significant predictors of major amputation [19]. In this context, it is worth mentioning that relevant factors for graft patency in oncovascular patients might differ from perioperative influences in patients with peripheral arterial occlusive disease (PAOD) [49]. Overall, prospective studies are needed to clarify the choice of vascular graft material in oncovascular surgery and to identify relevant cancer‐related influences on graft patency.

Consistent with recent studies on oncovascular and reconstructive procedures of STS and OGS, a wide range of graft patency and occlusion rates has been demonstrated in limb preservation. In general, reported postoperative graft occlusions ranged from 0% to 54% [12, 13, 18, 50]. These variations may be partly attributed to differences in anticoagulation protocols. Shah et al. reported a postoperative anticoagulation duration of 3 months. However, graft thrombosis occurred in 50% of implanted grafts (*n* = 8) [12]. In contrast, Tsukushi et al. employed a longer anticoagulation regimen of at least 6 months, similar to our own approach, and observed graft thrombosis in only 2 of 25 patients (8%) [11]. Nevertheless, the study by Tsukushi et al. reported a significantly higher rate of complications, including skin necrosis, infections, and pronounced edema, affecting 40% of patients.

In our cohort, perioperative complications were relatively low compared to other studies [7, 10, 11, 12]. Only one wound and graft infection occurred during the postoperative course, and operative and 30‐day mortality were zero. In contrast to other reports and clinical workflows, the interdisciplinary approach described here, involving oncologists, surgical pathologists, general, plastic, and vascular surgeons, may be the key to the effective treatment of patients with STS and OGS. In particular, the frequently described emergency surgery performed by called‐in and institutionally uninvolved vascular surgeons during oncovascular surgery has been completely avoided in the cohort described here [9]. Therefore, we suggest a specialization in high‐volume centers, clear clinical workflows, perioperative planning with the initial involvement of vascular surgeons, and the pursuit of an intersociety consensus and guidelines for treating patients with STS and OGS of the extremities. Moreover, the low prevalence of STS and OGS impedes the building and strengthening of study data and sufficient study cohorts. Therefore, the authors propose the setup of national or international register studies, which should include as many treated patients and outcome data as possible.

Finally, QoL is a mainly neglected aspect in the cohort described here, but extensive surgical treatment of the extremities has far‐reaching consequences for QoL, including serious problems with limited mobility and rehabilitation, which may lead to coping strategies, social isolation, and consequently higher morbidity and mortality [51, 52, 53]. To the best of the knowledge of the authors, there is still no standardized assessment of QoL in oncovascular patients. However, in conventional vascular medicine, there have been highly standardized solutions, for example, the Vascular Quality of Life Questionnaire‐6 (VascuQoL‐6) [54]. For future studies, it could be useful to adapt vascular‐related QoL assessments to oncovascular patients.

## 5 Conclusion

In summary, the treatment of patients with STS and OGS of the limbs and subsequent vascular reconstruction can be performed safely and effectively with acceptable surgical and oncological mid‐term results in terms of perioperative complications, vascular graft patency, and OS. The cohort described here and outcomes suggest that an interdisciplinary team, including vascular surgeons as well as an institutionalized and rigorous clinical proceeding, might positively influence the clinical and surgical outcomes. However, larger prospective studies are needed to better inform and guide both oncologists and surgeons in the future.

## Author Contributions

Conceptualization: Sebastian Kapahnke, Katharina Hess, Daniel Drücke, and Matthias Bürger. Methodology: Sebastian Kapahnke, Matthias Bürger, Philipp Johannes Pauli, Rouven Berndt, Martin Albrecht, Lars Hummitzsch, and Katharina Hess. Software: Sebastian Kapahnke and Matthias Bürger. Validation: Melanie Rusch, Grischa Hoffmann, Daniel Drücke, and Rene Rusch. Formal analysis: Sebastian Kapahnke, Daniel Drücke, and Matthias Bürger. Investigation: Sebastian Kapahnke, Matthias Bürger, Philipp Johannes Pauli, Roland Bertolini, Julia Bertolini, and Rouven Berndt. Resources: Sebastian Kapahnke, Matthias Bürger, Rouven Berndt, Daniel Drücke, and Katharina Hess. Data curation. Sebastian Kapahnke, Matthias Bürger. Writing – original draft preparation: Sebastian Kapahnke, Rouven Berndt, and Matthias Bürger. Writing – review and editing: Melanie Rusch, Grischa Hoffmann, Philipp Johannes Pauli, Lars Hummitzsch, Martin Albrecht, Julia Bertolini, Roland Bertolini, Rouven Berndt, Rene Rusch, Daniel Drücke, Christoph Röcken, Katharina Hess. Visualization: Sebastian Kapahnke, Matthias Bürger, Philipp Johannes Pauli, and Katharina Hess. Supervision: Rouven Berndt and Katharina Hess. Project administration: Rouven Berndt. All authors have read and agreed to the published version of the manuscript.

## Ethics Statement

The study was approved by the local ethics committee of the University Medical Center Schleswig‐Holstein, Kiel, Germany (protocol identification number: D463/24).

## Consent

All procedures, analyses, and data proceedings were performed in accordance with the Helsinki Declaration of 2013. The study was approved by the local ethics committee of the University Medical Center Schleswig‐Holstein, Kiel, Germany; protocol identification number: D463/24, which allowed us to use samples from those patients who had also given written informed consent for prospective scientific use of their patient material (broad consent).

## Conflicts of Interest

The authors declare no conflicts of interest.

## Data Availability

The data that support the findings of this study are available from the corresponding author upon reasonable request.
